# A situational analysis of latent tuberculosis infection among incarcerated population in Japan

**DOI:** 10.1371/journal.pone.0203815

**Published:** 2018-09-07

**Authors:** Lisa Kawatsu, Kazuhiro Uchimura, Akihiro Ohkado

**Affiliations:** Department of Epidemiology and Clinical Research, the Research Institute of Tuberculosis, Japan Anti- tuberculosis Association (RIT/JATA), Tokyo, Japan; Fundació Institut d’Investigació en Ciències de la Salut Germans Trias i Pujol, Universitat Autònoma de Barcelona, SPAIN

## Abstract

**Background:**

The World Health Organization recommends that systematic testing and screening of latent tuberculosis infection (LTBI) among the incarcerated population “should be considered”, though based on evidence of either low or very low quality. However, in Japan, a TB middle-burden country, systematic screening for LTBI in correctional facilities is currently not conducted. As part of a larger study to determine the cost-effectiveness of LTBI screening in correctional facilities in Japan, this study was conducted to determine the situation of LTBI, including treatment outcome, among the incarcerated population in Japan, and provide the essential data for cost-effectiveness analysis.

**Method:**

A cross-sectional study was conducted between 2017 and 2018 with public health centers which have one or more correctional facilities under their jurisdiction. Questionnaire surveys were sent to collect information on their policy of managing LTBI patients notified from correctional facilities, including whether or not there was a standardized procedure for initiating LTBI treatment, and also to collect sociodemographic information and treatment outcome of LTBI patients who were notified from the respective correctional facilities in 2015 and 2016.

**Results:**

The survey was sent to a total of 163 public health centers, out of which 133 (81.6%) responded. 8 of the 133 public health centers actively guided the correctional facilities regarding LTBI treatment initiation through a standardized procedure, while 115 either had not established such procedure or were unaware of how LTBI treatment was being initiated in the correctional facilities. A total of 91 LTBI patients were notified from the correctional facilities in 2015 and 2016, and the information of 89 were available for analysis. 82 were males, and 83 were Japan-born. Treatment outcome was known for 88 patients, of which 70 had completed treatment. Of the 18 who did not complete the treatment, 15 had been lost to follow-up upon release from the facilities. Among those who had been released whilst on treatment, the proportion of those who completed the treatment was higher in those patients who received pre-release visit by a public health nurse, than those who did not.

**Conclusions:**

LTBI treatment was often being initiated without consideration for the patients’ prison term. The treatment completion rate within jail was high, indicating the possibility that incarcerated population can benefit for LTBI treatment. On the other hand, the completion rate decreased significantly among those who had been released while still on treatment. In order to optimize the benefit, initiation of LTBI must carefully be considered upon the patient’s prison term, as well as coordination among the relevant organizations to ensure continuity of care after release.

## Introduction

It has been estimated that one third of the world’s population is infected with *M*.*tuberculosis* [1]. The lifetime risk of reactivation for tuberculosis (TB) among people with latent tuberculosis infection (LTBI) is estimated to be between 5 to 10%, with the majority developing the disease within the first five years after initial infection [2]. The End TB Strategy thus asserts that expansion of preventive treatment for people at high risk is an essential component of the global strategy for TB prevention, care and control beyond 2015 towards the elimination of TB [3]. The World Health Organization (WHO) similarly recommends active and systematic identification and treatment of people with LTBI for certain high–risk populations, mainly in high- and upper middle-income countries with an estimated TB incidence rate of less than 100 per 100,000 [4]. People incarcerated in correctional facilities are one such population, among who systematic testing and screening of LTBI “should be considered”, according to evidence of either low or very low quality [4].

In Japan, there are currently 187 correctional facilities, including prisons and jails, with an average daily incarcerated population of slightly less than 60,000 persons [5]. Health of the incarcerated population is under the jurisdiction of the Ministry of Justice, however, according to the Infectious Diseases Control Law of Japan, any physician who has diagnosed a case of TB or latent tuberculosis infection (LTBI) is required to notify the local public health center, and this applies to correctional physicians as well. It is then the responsibility of public health centers to enter patient information into the electronic national TB surveillance system, the Japan TB Surveillance (JTBS). The public health center also creates and maintains a case file in its local TB registry, with a more detailed information of the patients, collected either through interview with patients themselves, or communication with prison staff. The patient on the other hand may either be treated within his or her own facility, be sent to one of the prison hospitals, or at a non-prison hospital. When and if a patient is released while still on treatment, the correctional facility is advised to contact and coordinate with the public health center to arrange for post-release adherence support. However, although a guideline has been established regarding coordination between correctional facilities and public health centers in management of TB and LTBI among incarcerated populations, the actual practices still vary and are often influenced by personal relationships between the individual staff of public health centers and correctional facilities [6].

Using the publicly available data, we had previous estimated that TB notification among the incarcerated population may be up to ten times higher than that of the general population [7], which was 13.9 per 100,000 in 2016. Yet TB screening upon entry to correctional facilities is not conducted routinely, and chest X-ray is only mandatory in the annual health-check. Active LTBI screening in correctional facilities is not conducted systematically in Japan. As for treatment, in Japan, the Guideline on Treatment of LTBI [8], published by the Prevention Committee and the Treatment Committee of the Japanese Society for Tuberculosis, recommends 6- or 9-months regimen by isoniazid as first option, followed by 4- or 6-months regimen by rifampicin, which is only recommended when the possibility of the use of isoniazid is ruled out. However, the current guideline does not make any particular mention of preventive therapy for people who are incarcerated in correctional facilities, who may be at a higher risk of interrupting the treatment–especially if they are released while still on treatment. No study has so far been published on LTBI treatment regimen in correctional facilities in Japan and little is known about the detailed practices.

A project, funded by the Japan Society for the Promotion of Science is currently ongoing to determine the cost-effectiveness of screening for LTBI among the incarcerated population in Japan. Burden of LTBI and its treatment outcome among the incarcerated population is one of the key data needed to conduct the cost-effectiveness analysis–however, information regarding history of incarceration is not collected under the JTBS and thus it is not possible to determine the burden of LTBI or evaluate its treatment outcomes among the incarcerated population in Japan from the surveillance data. We thus conducted a survey of public health centers, whereby they were asked to retrieve information from their TB registries, with the purpose of determining the overview of the situation of LTBI, including treatment outcome, among the incarcerated population in Japan.

## Method

A two-step questionnaire survey, specifically designed for this study, was conducted with public health centers in Japan. The inclusion criteria for the first survey was all public health centers which have one or more correctional facilities under their jurisdiction. The survey was sent to all public health centers to investigate the number of LTBI notifications they received from correctional facilities between 1^st^ January 2015 and 31^st^ December 2016, and also to ask on management of LTBI in correctional facilities, especially regarding specific procedures, if any, for initiating preventive therapy for incarcerated persons. The inclusion criteria for the second survey was all those public health centers which have returned the first survey and which had notifications from the correctional facilities during the study period. The second survey included questions regarding demographic details of the patients, as well as clinical and treatment information and information regarding coordination with correctional facilities in ensuring continuation of care upon release. Public health centers which only reported receiving notifications for active TB were excluded. The data were entered into and managed using Excel (2013, Microsoft Corporation, US) and characteristics of the patients and treatment outcomes were analyzed, using R (version 3.1.3, R Development Core Team, Vienna, Austria). Characteristics of LTBI patients were compared with those of the newly incarcerated population in 2016, of which data was obtained from the Statistics of Correctional Facilities, compiled by the Ministry of Justice, Japan, and treatment outcomes were described. Proportions were compared using either chi-squared test or Fisher’s Exact test, as appropriate. Multiple regression was not conducted due to the small number of study population, and also large missing data for certain variables. No personal identifiers were collected, and thus informed consent was not obtained from the patients. The study protocol was reviewed and approved by the Institutional Review Board of the Research Institute of Tuberculosis, Japan Anti-Tuberculosis Association, Japan (reference number RIT/IRB 20–10).

## Results

### Procedures for initiating LTBI treatment

**Fig 1:**
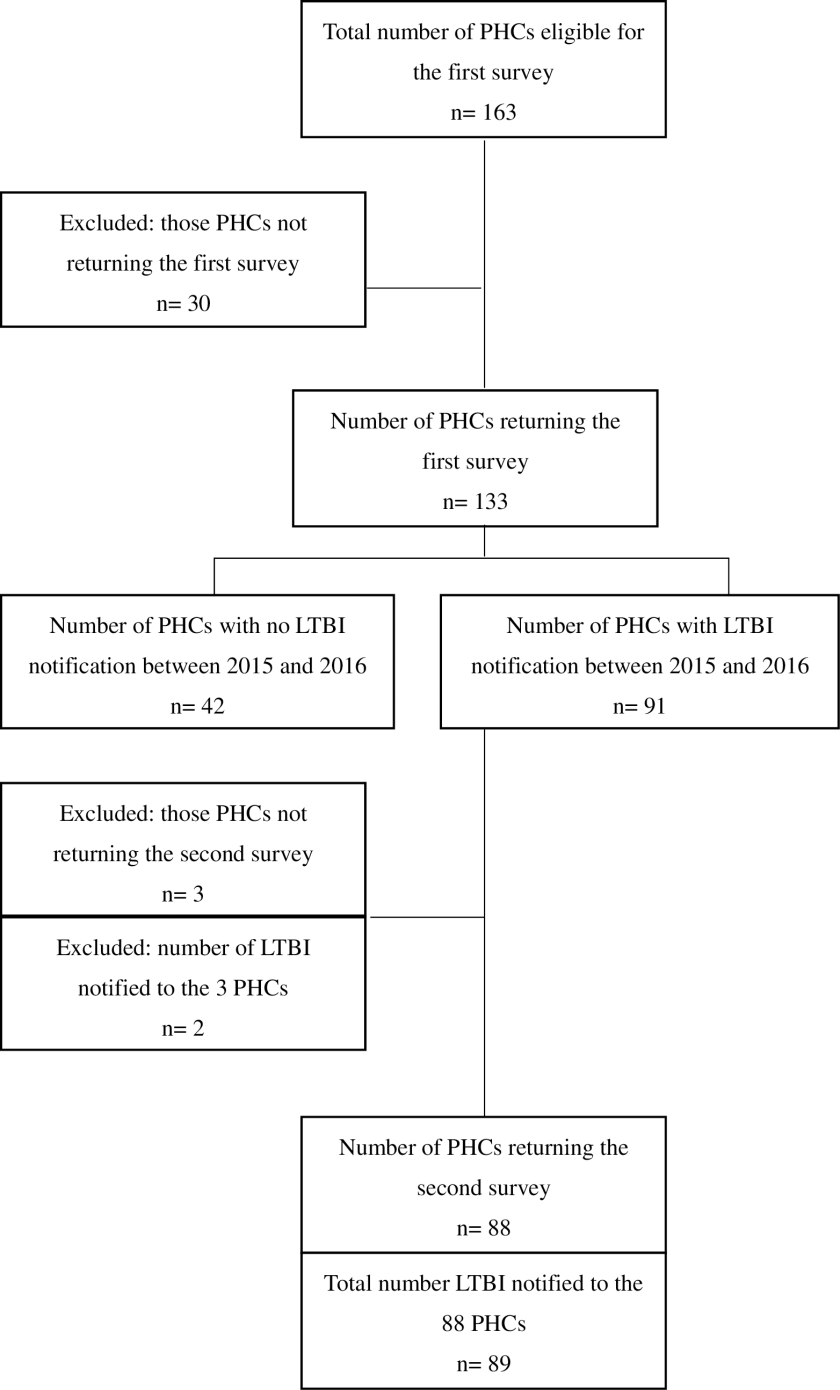
Flow chart of study population. PHC: public health center, LTBI latent tuberculosis infection.

Flow chart of the study population is shown in Fig 1.

The first survey was sent to a total of 163 public health centers, out of which 133 (81.6%) responded. The 133 public health centers covered 153 out of a total of 187 correctional facilities in Japan. Table 1 summarizes the types of correctional facilities which were covered in this study.

**Table 1:** Type of correctional facilities covered in this study.

Type of prison	n
General prison	62
Prison hospital	4
Jail	87
TOTAL	153

Regarding the procedures for initiating LTBI treatment for incarcerated persons, 8 (6.0%) of the 133 public health centers actively guided the correctional facilities regarding LTBI treatment initiation based on a standardized procedure, while 10 (7.5%) responded that they entrusted the decision to correctional facilities but were aware of how the decision was being made. The remaining 115 public health centers either did not have any standardized procedures, or were unaware of how LTBI treatment was being initiated in the correctional facilities. However, of the 8 public health centers which responded that they had a standardized procedure only two took into account the remaining duration of the patient’s prison term.

### Characteristic of notified LTBI patients

A total of 91 LTBI patients were notified from prisons and prison hospitals (i.e none from jails) in 2015 and 2016, to a total of 13 public health centers. However, 1 public health center failed to return the second survey, thus a detailed information of a total of 89 patients, notified to the remaining 12 public health centers, were available for analysis. 61.8% (n = 55) of the patients had been notified from one correctional facility, which had experienced a large outbreak and another 15.8% (n = 14) from another facility. The average number of notified case per facility, excluding the two facilities just mentioned, was 2 (range 1 to 5). 85 of the 89 patients had been notified as a result of contact investigation. Demographic characteristics of the 89 patients are summarized and compared with the newly incarcerated population in 2016, in Table 2. Information regarding treatment history, sources of infection and potential risk factors are summarized in Table 3.

**Table 2:** Characteristics of newly notified LTBI patients from correctional facilities, 2015–2016.

	LTBI patients		Newly incarcerated population in 2016	
	n	%	n	%
**Total**	89	100.0	20,467	100.0
**Sex**				
Male	82	92.1	18,462	90.2
Female	7	7.9	2,005	9.8
**Age group (years)**				
<20	0	0.0	30	0.1
20–29	2	2.2	2,840	13.9
30–39	23	25.8	4,715	23.0
40–49	27	30.3	5,596	27.3
50–59	30	33.7	3,536	17.3
60+	7	7.9	3,750	18.3
**Country of birth**				
Japan-born	83	93.3	19,723	96.4
Foreign-born	6	6.7	744	3.6

**Table 3:** Treatment history, sources of infection and possible risk factors of newly notified LTBI patients from correctional facilities, 2015–2016.

	n	%
**Total**	89	100.0
**Previous treatment history**		
None	86	96.6
Yes, for active TB	1	1.1
Yes, for LTBI	1	1.1
Unknown	1	1.1
**Sources of infection**		
TB patient of the same facility	82	78.3
TB patient of a different facility	1	6.6
TB patient with whom contact was made prior to entering the facility	4	4.7
Unknown	2	2.3
**Smoking prior to incarceration**		
Yes	8	9.0
No	3	3.4
Unknown	78	87.6
**Drug abuse prior to incarceration**		
Yes	13	14.6
No	3	3.4
Unknown	73	82.0
**Alcohol abuse prior to incarceration**		
Yes	1	1.1
No	7	7.9
Unknown	81	91.0
**Selected comorbidities**		
Hepatitis	16	18.0
Hypertension	11	12.4
Psychiatric disorders	7	7.9
Diabetes mellitus	6	6.7

TB: tuberculosis, LTBI: latent tuberculosis infection

82 (92.1%) were males, and 30 (33.7%) were aged 50–59 years. 83 (93.3%) were Japan-born. The proportion of males and of Japan-born were similar to (92.1% vs 90.2%, 93.3% vs 96.4%), however, the proportion of those aged 20–29 and 60 and above were smaller than, those of the newly incarcerated population in 2016 (2.2% vs 13.9%, 7.9% vs 18.3%).

86 (93.3%) were receiving treatment for the first time, however, two had history of previous treatment–one for active TB and another for LTBI. Sources of infection were known for 87 patients, of whom 82 were contacts of a TB patient from the same facility. Information regarding smoking, drug and alcohol abuse prior to incarceration was unknown for the majority of the patients. The most common comorbidity was hepatitis, reported in 16 (18.0%) patients, followed by hypertension (n = 11, 12.4%) and psychiatric disorders (n = 7, 7.9%).

### LTBI treatment status and outcome

All the 89 patients had initiated LTBI treatment at their respective facilities. Information regarding treatment regimen was known for 82 patients, of whom all were treated with isoniazid monotherapy. Of the 82 patients, 63 were treated with 6-months regimen, while the treatment duration was not known for the remaining 19 patients. Treatment outcome was known for 88 patients, and their characteristics are compared by treatment outcome in Table 4.

**Table 4:** Treatment outcome of LTBI by selected characteristics, 2015–2016 (n = 88).

	Completed (n = 70)		Not completed (n = 18)		Total (n = 88)	p-value
Sex	n	%	n	%		
Male	66	80.5	16	19.5	82	0.598
Female	4	66.7	2	33.3	6	
**Age group (years)**						
20–29	2	100.0	0	0	2	0.508
30–39	17	73.9	6	26.1	23	
40–49	22	84.6	4	15.4	26	
50–59	22	73.3	8	26.7	30	
60+	7	100.0	0	0	7	
**Country of birth**						
Japan-born	65	78.3	18	21.7	83	0.579
Foreign-born	5	100.0	0	0	5	
**Released while on treatment**						
Yes	22	59.5	15	40.5	37	<0.001
No	48	94.1	3	5.9	51	

LTBI: latent tuberculosis infection

Of the 88 patients, 70 (80.5%) had completed treatment. Of the 18 who did not complete the treatment, 15 had been lost to follow-up upon release from the facilities while still on treatment, one had been moved to another facility, one had died while on treatment, and the other had refused to continue taking the medication. There were no statistically significant differences in sex, age groups and country of birth between those who completed and did not complete LTBI treatment. However, there was a significantly higher proportion of those who had been released while on treatment among those who did not complete the treatment, compared with those who did (42.1% vs 2.0%).

Table 5 summarizes the 37 patients who were released while on treatment, by their treatment outcome, and whether any sort of coordination between the public health centers and the correctional facilities took place, prior to the patient being released.

**Table 5:** Treatment outcome of those who were released while still on LTBI treatment, 2015–2016 (n = 37).

	Completed (n = 22)		Not completed (n = 15)		Total (n = 37)	p-value
**Public health center informed of release date and patients’ contact details, prior to the release**	n	%	n	%		1.000
Yes	21	58.3	15	41.7	36	
No	1	100.0	0	0.0	1	
**Public health center nurse able to visit the patient and conduct health education, prior to release**						0.286
Yes	9	75.0	3	25.0	12	
No	13	52.0	12	48.0	25	

In all but one case, public health center was able to receive the basic details, including the date of release and contact details of the patient after he or she has returned to the community, from the correctional facilities prior to the actual release. Nevertheless, the proportions of those who did and did not complete the treatment were relatively similar (58.3% vs 41.7%). In 12 cases, the nurse of public health centers was able to meet the patient in person to give patient education prior to the release. The proportion of those who completed the treatment was higher than those patients who did not receive the visit by the public health center nurse (75.0% vs 52.0%), though the difference was not statistically significant.

## Discussion

This is the first study to have evaluated the situation of LTBI and treatment outcome among incarcerated population in Japan. The characteristics of the study participants were quite similar to the general incarcerated population in terms of sex and country of birth. However, the proportion of those aged 20–29, and aged 60 and above were smaller among the study participants. It is reasonable to see a smaller proportion of those aged 60 and above in the study participants, as LTBI treatment is not actively pursued for older patients in Japan from concerns for adverse events [8]. On the other hand, the study results could not provide a meaningful explanation as to why the proportion of those aged 20–29 was smaller among the study participants.

Despite the small sample size, the study results also provided important insight and highlighted two critical policy issues, namely, the need to make it a standard practice to consider the patient’s prison term in initiating LTBI treatment, and the need for a better coordination to ensure continuation of care after release. The aforementioned guideline for treatment of LTBI in Japan recommends 9 month-isoniazid regimen over the 6-month regimen, and the 4- or 6-months regimen by rifampicin is only recommended when the possibility of the use of isoniazid is ruled out [8]. Our results indicated that in fact, at least 71.6% (63/88) of the patients had received the 6-months regimen–however, the application of the 6-month regimen seemed not always be conditional upon the patient’s prison term as indicated from the results of the first survey. Thus, indeed, 42.0% (37/88) of the patients had been released while still on treatment, and the treatment completion rate was significantly lower among the latter, compared with those who completed the treatment within their prison term (57.9% vs 96.0%).

A systematic review of isoniazid preventive therapy in correctional facilities has reported the median completion rate of 44%, ranging from 3% to 87% [9]. Of the 18 studies included in the review, 7 reported on the completion rate within the correctional facilities, with the rate ranging from 31.6% [10] to 87.0% [11]. Compared with these results, the performance of LTBI treatment in the correctional facilities in Japan do appear to fare well. The completion rate was even higher than that of the general population, which has been reported to be around 70.0% [12], owing probably to the direct observed therapy being effectively implemented. Our results therefore suggest that incarcerated persons can and do benefit from LTBI treatment–which also has implications for discussions over LTBI screening–however, in order for the benefit to be optimized, best effort must be made to ensure completion, including considering the patient’s prison term prior to initiating the treatment and, which also leads to the second issue, coordinating to ensure continuation of care if and when patients are released while still on treatment.

In the abovementioned systematic review, the completion rates in studies which have followed-up patients after release have been disappointingly low, ranging from 6% to 60% [13,14]. In our study, though not statistically significant partially due to the small sample size, the proportion of those completing the treatment after release was higher in patients who received pre-release patient education by public health nurses than those who did not. However, the results of randomized controlled studies examining the effect of such intervention have been mixed. Two studies have reported that single pre-lease education and counselling did not improve post-release completion rates [15, 16], while others, albeit poor outcomes, concluded that more intentional sessions did improve the completion rate by two-fold [17,14]. Furthermore, several studies have suggested that post-treatment outcomes of infectious diseases, such as HIV, can be optimized with simultaneous treatment for substance abuse, which often co-exist with other health problems [18, 19]. In our study too, of the 16 patients whose history of substance abuse was known to the public health centers, 13 were recorded to be using illicit drugs prior to incarceration. A previous study has similarly reported high proportion of those with drug addiction among TB patients in the incarcerated population in Japan [20]. Exchanging and sharing basic patient information between the relevant organizations is necessary but insufficient on its own, and a more concerted and organized effort is necessary to ensure continuation of care and support for patients after they are released.

Finally, a note should be made of the potential for shorter regimens for LTBI treatment. In the early 2000s, a shorter regimen of 2-months rifampin and pyrazinamide (2RZ) had been recommended by the ATS/CDC [21] and several studies were conducted in the correctional facilities [22,23]. As compared with 6- or 12-months regimen by isoniazid, these studies showed significant improvement in the completion rate among those receiving shorter regimens. However, subsequent reports of severe hepatoxicity among HIV-negative recipients led to CDC amending the guideline for treatment for LTBI, preferring 9-months isoniazid regimen. In late 2011, the CDC recommended a new regimen of 3 months of weekly isoniazid and rifapentine (3HP) [24]. A systematic review with meta-analyses of efficacy and completion rates for shorter regimens, including the 3HP, concluded that these showed significant benefits in preventing active TB compared to placebos, and that regimens of 3 to 4 months were more likely to be associated with better completion [25]. Rifapetine is currently not approved in Japan, however, discussion are ongoing regarding its use [8].

This study is not without limitations. Due to the small sample size, we were limited to conducting minimal statistical analyses. Secondly, there was large missing data for several variables, which also precluded us from conducting additional analyses, including impact on treatment outcome. On the other hand, the status of data also revealed the difficulties public health centers continue to face in communicating with correctional facilities to collect the necessary patient information. Since the establishment of a guideline for public health centers on coordinating with correctional facilities in TB control in 2014, situation has certainly improved [6]. However, efforts must further be accelerated to strengthen coordination and collaboration among the relevant organizations in controlling TB and LTBI in correctional facilities in Japan.

## Conclusions

LTBI treatment was being initiated without systematic consideration for the patients’ prison term. The treatment completion rate within jail was high, indicating the possibility that incarcerated population can benefit for LTBI treatment. On the other hand, the completion rate decreased significantly among those who had been released while still on treatment. In order to optimize the benefit, initiation of LTBI must carefully be considered upon the patient’s prison term, as well as coordination among the relevant organizations to ensure continuity of care after release. Discussions over possibility of introducing shorter regimens for LTBI treatment and its potential benefit for population with higher risk of interrupting the treatment, such as persons released from correctional facilities, should be encouraged.

## Supporting information

S1 Table Raw data of the 88 LTBI patients, without the personal identifiers. (XLSX)
